# Research Progress of the Endocrine-Disrupting Effects of Disinfection Byproducts

**DOI:** 10.3390/jox12030013

**Published:** 2022-06-28

**Authors:** Shuxin Sui, Huihui Liu, Xianhai Yang

**Affiliations:** Jiangsu Key Laboratory of Chemical Pollution Control and Resources Reuse, School of Environmental and Biological Engineering, Nanjing University of Science and Technology, Nanjing 210094, China; suishuxin@njust.edu.cn (S.S.); hhliu@njust.edu.cn (H.L.)

**Keywords:** disinfection byproducts, endocrine-disrupting effect, adverse outcome pathways, molecular initiating event, receptor-mediated mechanism

## Abstract

Since 1974, more than 800 disinfection byproducts (DBPs) have been identified from disinfected drinking water, swimming pool water, wastewaters, etc. Some DBPs are recognized as contaminants of high environmental concern because they may induce many detrimental health (e.g., cancer, cytotoxicity, and genotoxicity) and/or ecological (e.g., acute toxicity and development toxicity on alga, crustacean, and fish) effects. However, the information on whether DBPs may elicit potential endocrine-disrupting effects in human and wildlife is scarce. It is the major objective of this paper to summarize the reported potential endocrine-disrupting effects of the identified DBPs in the view of adverse outcome pathways (AOPs). In this regard, we introduce the potential molecular initiating events (MIEs), key events (KEs), and adverse outcomes (AOs) associated with exposure to specific DBPs. The present evidence indicates that the endocrine system of organism can be perturbed by certain DBPs through some MIEs, including hormone receptor-mediated mechanisms and non-receptor-mediated mechanisms (e.g., hormone transport protein). Lastly, the gaps in our knowledge of the endocrine-disrupting effects of DBPs are highlighted, and critical directions for future studies are proposed.

## 1. Introduction

Disinfection processes, used for the public water system and aimed at inactivating viable pathogenic microorganisms and protecting against the occurrence of water-borne diseases, were considered as a significant public health triumph in the beginning of the 20th century [1,2,3]. However, it has been well demonstrated that several disinfection byproducts (DBPs) are unavoidably formed from the reaction between disinfectants and naturally organic matter, organic contaminants, or halides during water purification treatment [4,5]. Since the first group of DBPs, i.e., trihalomethanes (THMs), was found in 1974 [6], more than 800 DBPs belonging to various classes have been gradually determined both in real disinfection plants and in controlled laboratory tests [7,8]. With the development of analytical methods, it is conceivable that more DBPs will be continuously identified [9,10,11,12]. For example, Zhang, et al. [13] recently analyzed the DBPs in ozonated wastewater, and they identified eight new Br-DBPs, including 2-bromostyrene, 2-bromo-benzaldehyde, and 2-bromophenylacetonitrile. What are the potential harmful effects of the exposure of DBPs on human and wildlife?

It was reported that DBPs could enter organisms through a variety of exposure routes [10,14]. Individuals could intake DBPs not only through drinking water, but also via skin penetration and inhalation pathways when showering or swimming. DBPs have been detected in human biological matrices such as blood, urine, and alveolar air samples [15,16,17]. DBP exposure might adversely lead to health risks, including neurotoxicity, mutagenicity, teratogenicity, genotoxicity, developmental and reproductive issues, cytotoxicity, and carcinogenesis [4,18,19,20,21,22,23,24,25,26]. For instance, there is convincing evidence that exposure to THMs and haloacetic acids (HAAs) is associated with a high risk of bladder and colorectal cancer [4,27,28]. Recently, the results from epidemiological studies also implied that nitrogenous byproducts (haloamides, amines, halonitriles, and nitrosamines) may trigger bladder cancer [29]. To circumvent this health problem, the concentrations of a small fraction of DBPs, mainly THMs and HAAs in drinking water, are regulated by World Health Organization (WHO), United States Environmental Protection Agency, European Union, etc. [30,31,32]. However, recent studies revealed that commonly regulated DBPs cannot be the major contributor to the adverse health effects induced by consuming chlorinated drinking water [33,34]. Compared with regulated DBPs, some emerging DBPs, such as halobenzoquinones, iodinated DBPs, nitrogenous DBPs, and aromatic DBPs, are more worthy of attention [35,36].

Recently, concern has grown regarding the potential acute toxicity of certain wastewater-derived DBPs toward aquatic organisms. This concern is based upon the belief that the aquatic organisms may be exposed to increasing types of wastewater-derived DBPs because those compounds may enter the aquatic environment with the discharge of disinfected wastewater. To date, the potential acute toxicity of certain DBPs on typical aquatic organisms, such as alga, crustacean, and fish, has been reported [8,14,37,38,39,40,41,42,43,44,45,46,47]. For instance, we investigated the acute toxicity of seven wastewater-derived phenolic DBPs that belong to the typical five groups of phenolic DBPs (i.e., 2,4,6-trihalo-phenols, 3,5-dihalo-4-hydroxybenzaldehydes, 2,6-dihalo-4-nitrophenols, halo-salicylic acids, and 3,5-dihalo-4-hydroxybenzoic acids) toward *Gobiocypris rarus* and found that the half lethal concentration (LC_50_) values of 2,4,6-trihalo-phenols and 2,6-dihalo-4-nitrophenols was in the 1–10 mg/L range, indicating that their acute toxicity should not be neglected [48]. In addition to the aforementioned health and ecological effects, can DBPs elicit other potential adverse effects, such as endocrine-disrupting effects?

The endocrine hormones of organisms such as thyroid hormones (THs), estrogen, and androgen regulate many critical physiological processes, e.g., growth and metamorphosis [49,50,51]. However, it is well known that a number of anthropogenic substances named endocrine-disrupting chemicals (EDCs) can elicit potential endocrine-disrupting effects on human and wildlife [52,53,54]. In this regard, EDCs are recognized as a serious threat to human health and the environment. In order to minimize the adverse health and environment impacts of EDCs, it is urgent to identify and screen potential EDCs from artificial chemical substances and unintentional production chemicals (e.g., DBPs) [55]. Actually, despite more than 800 individual DBPs having been identified in previous studies, only a few have been assessed for their potential endocrine-disrupting potency. Recently, Gonsioroski et al. reviewed the adverse reproductive effects in nonhuman animals and humans for some groups of EDCs in water such as DBPs, fluorinated compounds, bisphenol A, phthalates, pesticides, and estrogens [26]. However, it deserves mention that no comprehensive information related to the potential endocrine-disrupting effects of DBPs in the view of adverse outcome pathways (AOPs) is available up to now. Thus, it is significant to clarify which types of DBPs can exhibit endocrine-related detrimental effects, and which endocrine-related targets can be disturbed by DBPs.

Here, we attempted to present a significant overview of the potential endocrine-disrupting effects of DBPs in the view of AOPs on the basis of data derived from the published literature. The aim of this work was to (1) provide an updated, systematic and comprehensive review on the aspects of molecular initiating events (MIEs) disturbed by DBPs, (2) review the underlying toxicological key events (KEs) of DBPs, and (3) present adverse outcomes (AOs) of DBPs in mammals and aquatic vertebrates.

## 2. Performance of Publications

**Figure 1:**
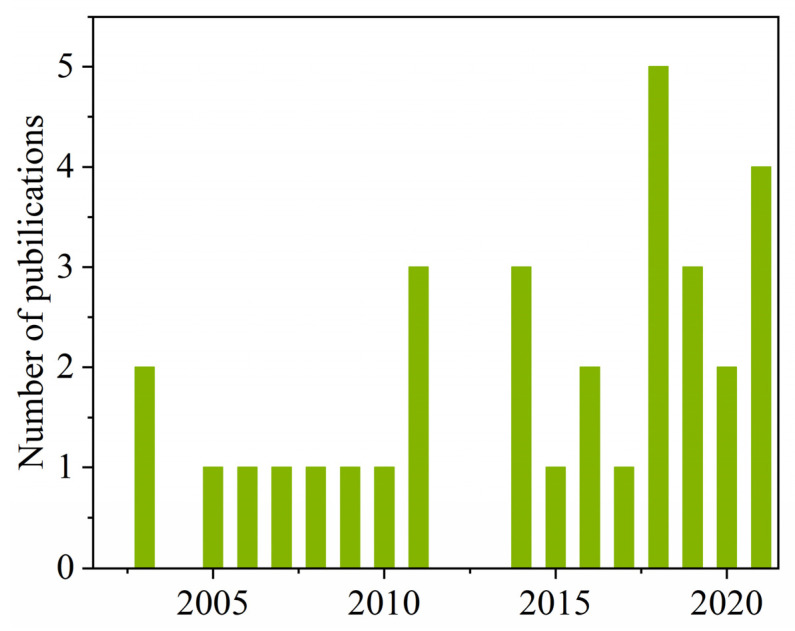
The number of publications on endocrine-disrupting DBPs from 2000 to 2022.

The endocrine-disrupting data of disinfection byproducts referred to in this study were obtained from published papers identified in the database of Web of Science (www.isiwebofknowledge.com, accessed on 7 January 2022) within the years 2000 to 2022. The search terms were “disinfection byproducts” and “endocrine”. The available literature was further refined by considering whether MIEs were defined or not. Finally, 32 studies related to the endocrine system-disrupting effects of DBPs were selected in the present investigation [28,49,50,51,56,57,58,59,60,61,62,63,64,65,66,67,68,69,70,71,72,73,74,75,76,77,78,79,80,81,82,83]. As expected, most of the research on the endocrine-related detrimental effects of various DBPs were published in the last ten years even though the first publication dated back to 2003 (only seven publications from 2000 to 2009 and 25 from 2010 until now) (Figure 1). This means that the endocrine-perturbing effects of DBPs have gradually attracted people’s attention. In total, 131 DBPs and 14 endocrine-related targets were summarized from these studies. Detailed information of the studies, DBPs, and endocrine-related targets is listed in Supplementary Table S1.

## 3. Characterization of DBPs with Endocrine-Disrupting Data

**Figure 2:**
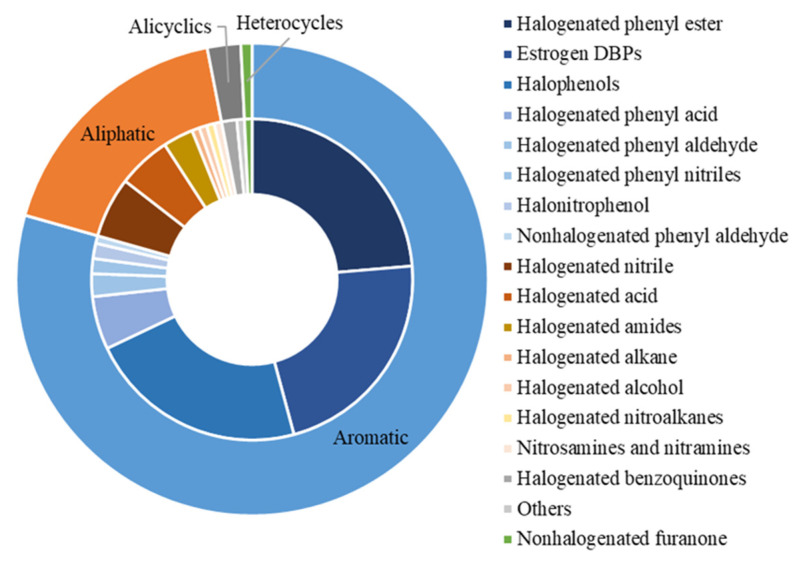
Summary of DBPs identified with endocrine-disrupting potential.

As shown in Figure 2, these 131 DBPs could be divided into four classes (i.e., aromatic, aliphatic, alicyclic, and heterocyclic DBPs) on the basis of their chemical structure. Aromatic DBPs could be further classified into eight subgroups (i.e., halogenated phenyl esters, estrogen DBPs, halophenols, halogenated phenyl acids, halogenated phenyl aldehydes, halogenated phenyl nitriles, halonitrophenols, and nonhalogenated phenyl aldehydes). Aliphatic DBPs included seven subgroups (i.e., halogenated nitriles, halogenated acids, halogenated amides, halogenated alkanes, halogenated alcohols, nitrosamines and nitramines, and halogenated nitroalkanes). Alicyclic DBPs contained two subgroups (halogenated benzoquinones and others). Heterocyclic DBPs were represented by nonhalogenated furanone.

The studied endocrine endpoints, as well as the corresponding DBP subgroups, are listed in Table 1. As shown, several DBPs in each studied subgroup except for halogenated phenyl esters and estrogen DBPs were investigated for their potential endocrine-disrupting effects. For halogenated phenyl esters and estrogen DBPs, however, more than 20 substances for each subgroup were tested for their potential activating/inhibiting potency toward human estrogen receptor α (hERα) and human aryl hydrocarbon receptor (hAhR). In addition, special attention was given to whether halophenols may pose a hazard to the endocrine system of organisms. For example, 12 out of 14 studied endocrine endpoints were tested using halophenols as model compounds. We also found that at least four subgroups of DBPs were evaluated for their potential interactions with hERα, human androgen receptor (hAR), and human transthyretin (hTTR).

**Figure 3:**
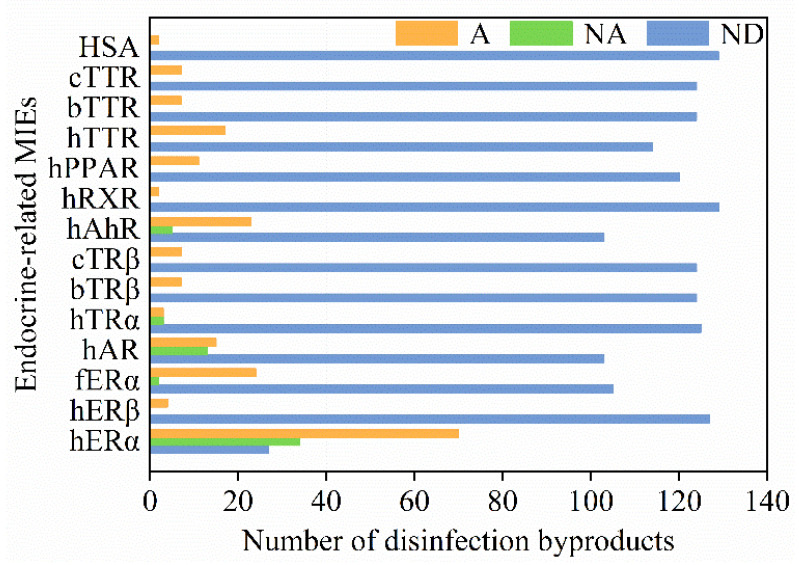
Overview of DBPs and their associated endocrine-disrupting effects. Orange, the number of active compounds (A); green, the number of inactive compounds (NA); blue, the number of compounds without available data (ND). Abbreviations: hERα—human estrogen receptor α; hERβ—human estrogen receptor β; fERα—medaka fish estrogen receptor α; hAR—human androgen receptor; hTRα—human thyroid receptor α; bTRβ—bullfrog thyroid receptor β; cTRβ—chicken thyroid receptor β; hAhR—human aryl hydrocarbon receptor; hRXR—human retinoic X receptor; hPPAR—peroxisome proliferator–activated receptor; hTTR—human transthyretin; bTTR—bullfrog transthyretin; cTTR—chicken transthyretin; HSA—human serum albumin.

For each studied endocrine endpoint, we also summarized the number of active compounds, inactive compounds, and compounds without available data. As shown in Figure 3, the number of active compounds for hERα was more than that of other endpoints. For hERα, human aryl hydrocarbon receptor (hAhR), and human androgen receptor (hAR), the number of active compounds was greater than that of inactive compounds. On the other hand, all the tested DBPs were active compounds for human transthyretin (hTTR), bullfrog transthyretin (bTTR), chicken transthyretin (cTTR), human serum albumin (HSA), peroxisome proliferator–activated receptor (hPPAR), human retinoic X receptor (hRXR), bullfrog thyroid receptor β (bTRβ), chicken thyroid receptor β (cTRβ), and human estrogen receptor β (hERβ). 

## 4. Endocrine-Related MIEs of DBPs

**Figure 4:**
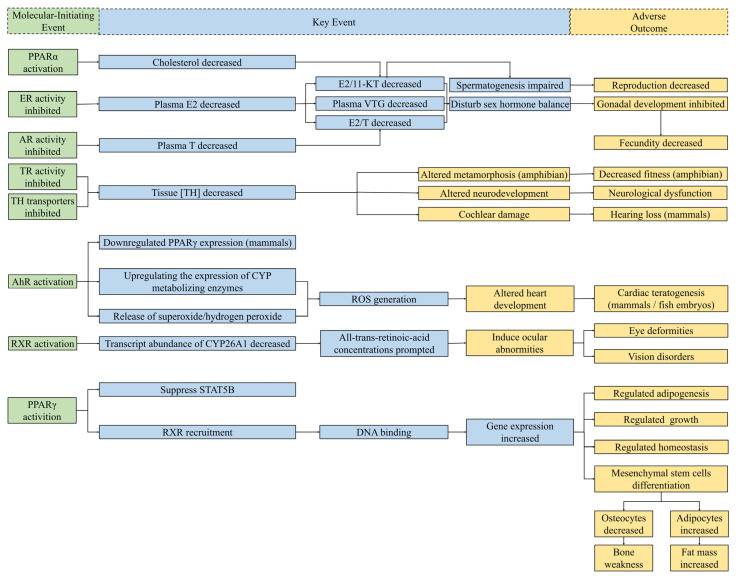
Schematic diagram of known MIEs for endocrine disruption of DBPs and several related potential KEs and AOs. The relationships between MIEs and potential KEs in this figure were collected from compounds other than DBPs [86,87,88,89,90,91]. Considering the significant relationships between each step, MIEs (**left**) bring about KEs (**middle**), and then lead to AOs (**right**). Green boxes, MIEs associated with endocrine perturbation of DBPs; blue boxes, KEs of endocrine disruptors collected from studies; yellow boxes, AOs for endocrine toxicity. Abbreviations: E2, estradiol; T, testosterone; 11-KT, 11-ketotestosterone; VTG, vitellogenin; TH, thyroid hormones; CYP, cytochrome P450; ROS, reactive oxygen species; STATSB, signal transducer and activator of transcription 5B.

DBPs can disturb normal endocrine homeostasis by regulating the hormone system for fundamental physiological and developmental control [84]. The perturbing mechanisms of DBPs include activating/inhibiting nuclear receptors and interfering with non-receptor-mediated pathways. It is reported that most of adverse outcomes of endocrine-disrupting chemicals (EDCs) are attributed to the fact that they interfere with nuclear receptor (NR)-mediated hormone signals [60]. The substance structure of some DBPs is similar to that of natural hormones; thus, they can directly bind with receptors, interfere with the hormone pathway, and show distinct disrupting activities. The mediated physiological and biochemical pathways of several receptors on which the Guidance for the Identification of Endocrine Disruptors (EFSA/ECHA, 2018) focuses [85], including androgen receptor (AR), estrogen receptor (ER), and thyroid receptor (TR), are of critical importance in significant biological studies of endocrine disruption effects. All the tested molecular-initiating events related to DBPs are illustrated in Figure 4.

### 4.1. Hormone Receptor-Mediated Mechanism of Endocrine Disruption

Estrogen receptors (ERs) have critical roles in the growth and development of organisms [53]. The recombinant yeast screening bioassay, the E-screen assay of MCF-7 and MVLN cell line, and the uterotrophic bioassay are usually adopted for identifying potential estrogenic disruptors [51,71,83]. Our analysis results indicated that 70 DBPs have been proven to have estrogenic activity, i.e., they can interfere with ER. There is evidence in toxicological and epidemiological research in cell cultures that haloacetonitriles (HANs), e.g., dibromoacetonitrile (DBAN) and 2,3-dibromopropionitrile (DBPN), can invoke adverse effects on the endocrine system by binding to the human estrogen receptor and androgen receptor [50,66]. Additionally, Nakamura et al. [58] reported that halogenated derivatives of E1, E2, E3, and EE2 showed estrogenic activity, interfering with estrogen receptor α, using yeast two-hybrid assays between human and medaka fish (*Oryzias latipes*), and the ER-binding potency of halogenated DBPs of estrogens substituted at the 2- and 4-positions displayed a similar trend.

The androgen hormone regulates the androgen signaling pathway via binding with the androgen receptor (AR), and it plays an essential role in the physiological processes of human development and reproduction [92]. Iodoacetic acid (IAA) was observed to show AR binding in vitro [50]. Despite the discrepancies between this result and others, studies have still demonstrated that IAA is a potential disruptor of human AR (hAR) [51]. The differences in research results may be due to factors such as the selection of species of cells and diverse endpoints. Additionally, among haloacetamide DBPs, bromoacetamide (BAM) exhibited slight androgenic activity according to a yeast-based reporter bioassay [69]. Notably, iodoacetonitrile (IAN) generated from water disinfection processes was found to have a weak androgenic effect (11.4% induction) at the highest concentration [71].

Thyroid hormones (THs), a series of essential endocrine hormones, are synthesized and secreted by thyroid follicular cells. They exist in many tissues in the brain, heart, liver, etc., where they regulate metabolism and development [82]. THs, especially triiodothyronine (T3), mainly moderate gene transcription or protein expression via binding to thyroid hormone receptors (TRs) [93]. Halogenated derivatives of bisphenol A (BPA) have been shown to act as agonists/antagonists for TH receptors, affecting the levels of THs and invoking thyroid system disruption in organisms. 3,3’,5,5’-Tetrabromobisphenol A (TBBPA), 3,3’,5,5’-tetrachlorobisphenol A (TCBPA), and 3,3’,5-trichlorobisphenol A (3,3’,5-triClBPA) were proven to possess human TH agonist activity in a yeast two-hybrid assay incorporating hTRα [59]. In addition, Yamauchi et al. [56] investigated the influence of chlorinated compounds of BPA on T3 binding with the TR ligand-binding domains between chicken and bullfrog but demonstrated that they were unlikely to be TH system-disrupting compounds for these animals.

Some chemicals could bind to other receptors to indirectly participate in hormone regulation instead of acting directly on hormone receptors. For example, peroxisome proliferator-activated receptor gamma (PPARγ), expressed in the fatty tissue, is a critical transcription element in the development and metabolism of adipocytes [65]. The imbalance of PPAR might be associated with diseases such as diabetes, obesity, and dysgenesis [94]. A previous 293T cell-based luciferase reporter bioassay indicated that chlorinated BPS analogs enhanced PPAR activities as opposed to the parent compound, and their activities were correlated to the values of log*K*_ow_ [65]. TBBPA and TCBPA could also activate PPAR through direct interaction with humans or animals, and the activation potential highly relied on the halogenation degree [60,61]. The results from in vitro experiments revealed that halogenated products of BPF were also potential disruptors of PPAR, similar to those of BPA and BPS [64]. Taken together, the presence of DBPs of BPA, BPS, and BPF in disinfected water should be of concern because they could pose a potential risk to mitigation of inflammation.

Furthermore, human retinoic X receptor (RXRs) have also been shown to be endocrine-related targets for DBPs action. RXRs are key partners for the nuclear receptor signaling pathways of cell growth, differentiation, and metabolism [95]. Chlorination byproducts of BPA have been identified as RXRβ antagonists, the antagonist activities of which are much higher than that of BPA according to a yeast assay [63]. Considering that previous studies documented that BPA could exhibit several detrimental effects (e.g., endocrine-related harmful effects) on organisms [96,97,98,99,100], those results indicate that both BPA and its halogenated DBPs are potential endocrine disruptors. Experimental evidence for DBPs with respect to their AhR binding affinities is rather limited. In terms of structure, halogenated parabens are similar to halogenated aromatic hydrocarbons, which were determined to possess AhR potency. Experimental values obtained via a yeast bioassay and HepG2 cells showed that the AhR activity of monochlorinated parabens was more effective than that of their unsubstituted or chlorinated counterparts [62]. Analogously, this regular pattern is also applicable to monobrominated by-products. Promisingly, it was noted that 3-BrBP, 3-BrBnP, and 3-BriBP, compared with their unsubstituted and brominated corresponding counterparts, were proven to have the highest AhR activity with *EC*_50_ values of 3.9 nM, 9.0 nM, and 9.6 nM, respectively [70].

### 4.2. Non-Receptor-Mediated Mechanism of Endocrine Disruption

It has been recognized that activation or inhibition of nuclear receptors is not the only endocrine-disrupting pathway for DBPs to exert endocrine-perturbing effects [85]. Another toxicity pathway leading to an endocrine-related detrimental influence is the non-receptor-mediated mechanism [69]. Instead of acting directly on nuclear receptors, the pathway of non-receptor-mediated activity interference comprises inhibition of protein synthesis, destruction of β-galactosidase gene transcription, and inhibition of enzyme activity [101]. Endocrine disruptors can affect some links of the hypothalamus–pituitary–thyroid (HPT), hypothalamic–pituitary–gonadal (HPG), and hypothalamic–pituitary–adrenal (HPA) axes, and further disturb hormones biosynthesis, secretion, transport, metabolism, and feedback regulation [89,102]. There are three transporters in human blood that carry THs to target tissues: transthyretin (TTR), thyroxine-binding globulin (TBG), and albumin (ALB) [103].

The results from Yang et al. [67] revealed that 2,4,6-trihalo-phenols, 2,6-dihalo-4-nitrophenols, and 3,5-dihalo-4-hydroxybenzaldehydes, representing emerging polar phenolic DBPs, were identified as high-potency binders to compete with THs for binding to human TTR. Disrupting the transportation of TH might bring about DBPs being delivered to unexpected sites, which might further induce TH-related perturbing effects [102,104]. Previous evidence also showed that 2,6-dichloro-4-nonylphenol is a potent competitor of T3 interacting with chicken and bullfrog TTR, along with by-products of nonylphenol [56]. Furthermore, the comparison of TTR-binding activities among brominated derivatives of BPA indicated that the presence of a hydroxyl group at the *para* position and halogen substituents were conditions for TTR-binding effects [105]. These experimental results may confirm the conclusion that halogenated aromatic chemicals with phenol hydroxy groups can be considered as binders to TTR owing to their similar structure to the natural thyroxine (T4) [106]. ALB is also a potential endocrine-related target in the mechanism of TH transport disruption. According to competitive binding assays, 4-bromophenol and 2,4-dibromophenol were observed to interfere with human serum albumin (HSA) to form complexes [68]. Remarkably, 2,4-dibromophenol had a high binding affinity to HSA.

## 5. Potential Endocrine Adverse Outcome Pathways of DBPs

Compared with studies about molecular-initiating events, only a few studies focused on revealing the potential endocrine-related key events and adverse outcomes after DBP exposure. An in vivo experiment indicated that IAA, an aliphatic DBP, increased the weight of the testes of parental male rats and shortened the anorectal distance of male pups [51]. However, the specific toxicity mechanisms remain unclear and require to be further confirmed. Additionally, IAA exposure reduced the level of triiodothyronine (T3), but upregulated the thyrotropin-releasing hormone level and thyrotropin level, which could also result in changes in the thyroid follicles of Sprague-Dawley (SD) rats [82]. The possible molecular mechanism of thyroid gland function disruption might be associated with the binding potency of nuclear receptors. In vivo toxicity reports demonstrated that histopathological changes in both heart and brain induced by 2,6-dichloro-1, 4-benzoquinone exposure for adult zebrafish could be attributed to oxidative stress [107]. The results from in vivo experiences showed that bisphenol S disinfected derivatives could influence the mRNA expression level of TRβ in zebrafish larvae [49], which could further mediate the bioactivities of thyroid hormone. Additionally, limited toxicological reports in vivo revealed no significant indication for plasma VTG levels in adult *Danio rerio* during 21 day exposure to TBBPA and TCBPA disinfection derivatives [83]. The developmental toxicity induced by TCBPA and TBBPA disinfection derivatives might be irrelevant to their estrogenic activities. In vivo assays of estrogenic activity showed that 3-chlorobisphenol A and 3,3’-dichlorobisphenol A each evidently enlarged the uterine endometrial area in rats treated with 100 mg/kg/day of these substances [57]. Wang et al. [108] linked the developmental toxicity of halobenzoquinone to oxidative stress, but they did not link the ROS generation with MIEs of endocrine disruption. The relationship between endocrine-related MIEs and oxidative stress was revealed in animal toxicity studies showing that aryl hydrocarbon receptor (AhR) activation could increase ROS generation by regulating the expression of Cyp1b1, which led to cardiac malformation in zebrafish embryos [86].

## 6. Conclusions and Future Directions

With the development of analytical methods, a large number of DBPs are being continuously detected and identified in treated drinking water, purified swimming pool water, disinfected wastewater, etc. Here, we summarized the literature on the endocrine-disrupting effects of DBPs. The results from the limited studies suggested that exposure to some DBPs could elicit endocrine-related detrimental effects not only on humans, but also on other wildlife, e.g., aquatic vertebrates. Our analysis results also revealed that the available data related to the potential endocrine system-disrupting properties of DBPs are limited to molecular-initiating events, i.e., biomacromolecules in the endocrine system. The identified molecular-initiating events mainly involved receptor-mediated toxicity pathways.

The future directions are proposed below.

(1) Development of appropriate screening strategy for assessing the potential endocrine-disrupting effects of DBPs

It was reported that the cost to evaluate the potential endocrine-disrupting effects of one substance is about 1 million USD [109]. In this case, it is impossible to screen the potential EDCs from more than 800 identified DBPs using experimental assays only. Considering that computational models are cost-effective and rapid methods, a comprehensive screening strategy containing both computational models and experimental assays should be employed to identify the potential EDCs from analyzed DBPs. In this comprehensive screening strategy, the endocrine-related computational models can be firstly used to set the priority. Then, the limited test resources can be focused on verifying whether the DBPs with high priority are endocrine disruptors or not.

(2) Clarifying the potential endocrine-related adverse outcome after DBP exposure

In addition to revealing the endocrine-related molecular-initiating events influenced by DBP exposure, further biological studies are expected to illustrate the potential endocrine-related key events and adverse outcomes following DBP exposure, as well as confirm the detailed relationship of molecular-initiating events with key events and adverse outcomes.

(3) Attention to non-receptor-mediated toxicity pathways

In addition to the receptor-mediated model of action, EDCs may perturb the endocrine system via a non-receptor-mediated mode of action, such as by interfering with targets related to biosynthesis and metabolism and plasma binding. In future studies, we should pay more attention to testing the potential non-receptor-mediated toxicity pathways of DBPs.

## Figures and Tables

**Table 1:** Summary of all groups of DBPs focusing on endocrine activity.

Endpoints	Groups of DBPs
hERα	Halogenated phenyl esters (31); estrogen DBPs (29); halophenols (14); halogenated nitriles (8); halogenated acids (7); halogenated amides (4); halogenated phenyl nitriles (2); halogenated benzoquinones (2); halogenated alcohols (1); halogenated alkanes (1); halogenated nitroalkanes (1); nitrosamines and nitramines (1); nonhalogenated phenyl aldehydes (1); nonhalogenated furanone (1); others (1)
hERβ	Halophenols (4)
fERα	Estrogen DBPs (4)
hAR	Halogenated nitrile (8); halogenated acids (7); halogenated amides (4); halophenols (2); halogenated benzoquinones (2); halogenated alcohols (1); halogenated alkanes (1); halogenated nitroalkanes (1); nitrosamines and nitramines (1); nonhalogenated furanone (1)
hTRα	Halophenols (6)
bTRβ	Halophenols (7)
cTRβ	Halophenols (7)
hAhR	Halogenated phenyl ester (28)
hRXR	Halophenols (2)
hPPAR	Halophenols (11)
hTTR	Halogenated phenyl acid (7); halophenols (5); halogenated phenyl aldehydes (3); halonitrophenols (2)
bTTR	Halophenols (7)
cTTR	Halophenols (7)
hHSA	Halophenols (2)

Note: Numbers in brackets represent the total number of DBPs studied for potential endocrine-disrupting effects in each group. Abbreviations: hERα—human estrogen receptor α; hERβ—human estrogen receptor β; fERα—medaka fish estrogen receptor α; hAR—human androgen receptor; hTRα—human thyroid receptor α; bTRβ—bullfrog thyroid receptor β; cTRβ—chicken thyroid receptor β; hAhR—human aryl hydrocarbon receptor; hRXR—human retinoic X receptor; hPPAR—peroxisome proliferator–activated receptor; hTTR—human transthyretin; bTTR—bullfrog transthyretin; cTTR—chicken transthyretin; HSA—human serum albumin.

## Data Availability

Not applicable.

## Supplementary Materials

The following supporting information can be downloaded at https://www.mdpi.com/article/10.3390/jox12030013/s1: Table S1. Available disinfection byproducts and endocrine-related targets.
